# Supplementary material for: Testing Two Somatic Cell Count Cutoff Values for Bovine Subclinical Mastitis Detection Based on Milk Microbiota and Peripheral Blood Leukocyte Transcriptome Profile
Source: Animals (Basel). 2022 Jun 30;12(13):1694. doi: 10.3390/ani12131694 (PMC9264859; doi:10.3390/ani12131694)
Supplement: Supplementary file 1 [file animals-12-01694-s001.zip › animals-1758589-supplementary.pdf]

Supplementary Table S1. 16S rRNA gene V3-V4 amplification PCR reactants

Reactants	dosage
High-Fidelity PCR Master Mix	15 μL
Forward primers	0.2 μM
Reverse primers	0.2 μM
DNA template	10 ng

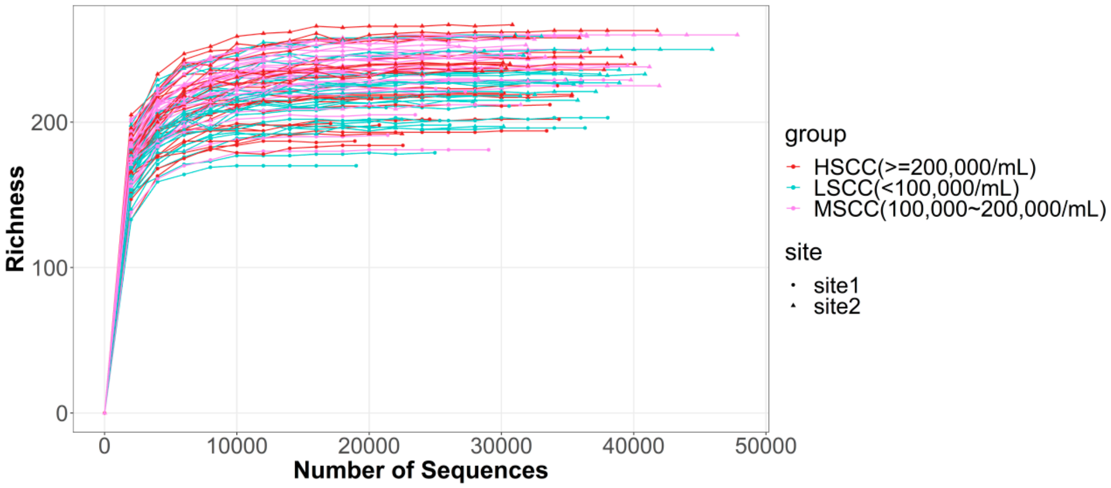

Supplementary Figure S1. Rarefaction curves at the genus level

Supplementary Table S2. Pairwise comparison of alpha diversity after correcting covariates

Comparison	P value				
	Shannon	Simpson	Inverse Simpson	Chao1	ACE
HSCC *vs.* LSCC	0.43	0.31	0.30	0.67	0.69
HSCC vs. MSCC	1	0.92	0.97	0.58	0.56
LSCC vs. MSCC	0.49	0.54	0.43	0.16	0.16

Significance was determined at P <0.05. LSCC: low SCC group (SCC < 100,000 cells/ml), MSCC: medium SCC group (100,000 cells/ml ⩽ SCC ⩽ 200,000 cells/ml), HSCC: high SCC group (SCC ⩾ 200,000 cells/ml).

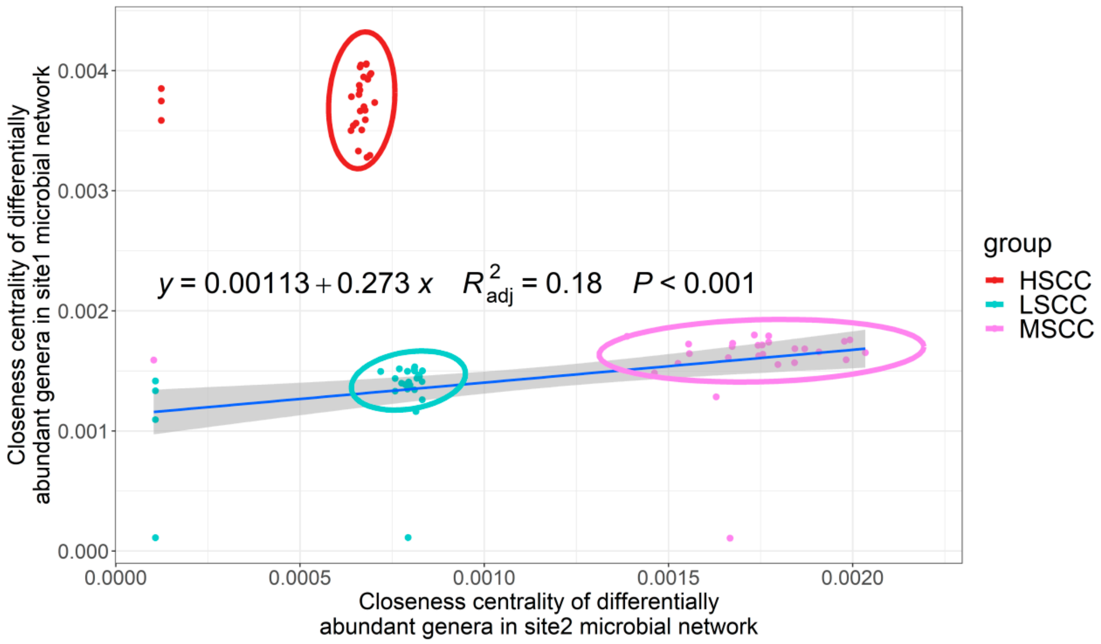

Supplementary Figure S2. Closeness centrality of differentially abundant genera shared

in two sites. Linear regression analysis was conducted on the closeness centrality of shared differentially abundant genera calculated in MSCC and LSCC network.

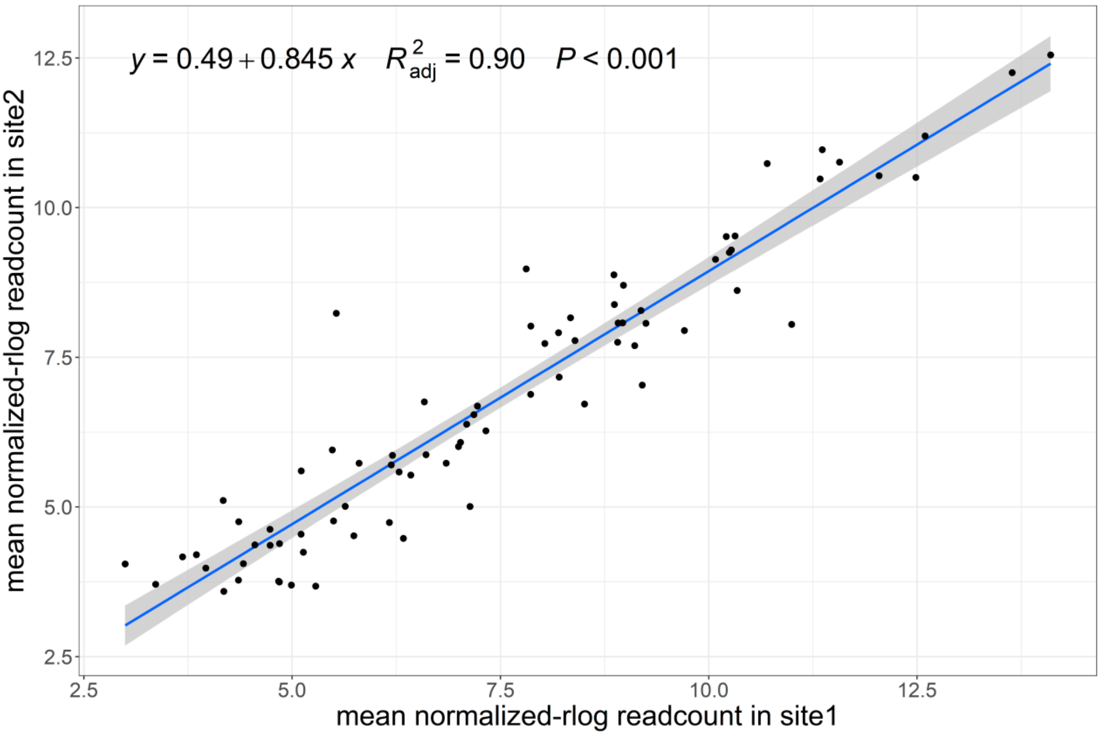

Supplementary Figure S3. Linear regression analysis of expression levels of shared DEGs with the same regulation direction in two sites
